# Detecting Fat Content of Food from a Distance: Olfactory-Based Fat Discrimination in Humans

**DOI:** 10.1371/journal.pone.0085977

**Published:** 2014-01-22

**Authors:** Sanne Boesveldt, Johan N. Lundström

**Affiliations:** 1 Division of Human Nutrition, Wageningen University, Wageningen, the Netherlands; 2 Monell Chemical Senses Center, Philadelphia, Pennsylvania, United States of America; 3 Department of Clinical Neuroscience, Karolinska Institutet, Stockholm, Sweden; 4 Department of Psychology, University of Pennsylvania, Philadelphia, Pennsylvania, United States of America; Duke University, United States of America

## Abstract

The desire to consume high volumes of fat is thought to originate from an evolutionary pressure to hoard calories, and fat is among the few energy sources that we can store over a longer time period. From an ecological perspective, however, it would be beneficial to detect fat from a distance, before ingesting it. Previous results indicate that humans detect high concentrations of fatty acids by their odor. More important though, would be the ability to detect fat content in real food products. In a series of three sequential experiments, using study populations from different cultures, we demonstrated that individuals are able to reliably detect fat content of food via odors alone. Over all three experiments, results clearly demonstrated that humans were able to detect minute differences between milk samples with varying grades of fat, even when embedded within a milk odor. Moreover, we found no relation between this performance and either BMI or dairy consumption, thereby suggesting that this is not a learned ability or dependent on nutritional traits. We argue that our findings that humans can detect the fat content of food via odors may open up new and innovative future paths towards a general reduction in our fat intake, and future studies should focus on determining the components in milk responsible for this effect.

## Introduction

In many Western diets, up to 40% of daily caloric intake is in the form of lipids, despite the fact that the recommended level for most individuals is at least 10% lower [1]. The desire to consume high volumes of fat is thought to originate from an evolutionary pressure to hoard calories, and fat is among the few energy sources that we can efficiently store over a longer time period. Indeed, recent data indicate that the human oral cavity contains putative taste receptors that are specifically tuned to recognize fatty acids [2], [3]. Moreover, behavioral studies have provided evidence for oral chemosensory detection mechanisms for various types of free fatty acids [4]–[6], and have shown that humans have different detection thresholds for different routes of stimulus presentation [7]. This physiological system would be an evolutionary benefit in times of food scarcity.

From an ecological perspective, however, it would be clearly advantageous to detect the fat content of food from a distance in order to maximize the chances of finding a source of calories, and before a potentially toxic substance enters the mouth for nutrient evaluation. Support for this basic assumption already comes from animal literature in which the role of olfaction in the preference for fat foods and triglycerides has been clearly established [8]–[11]. Moreover, Halpern and colleagues recently demonstrated that humans can discriminate various vapor-phase long-chain fatty acids (linoleic, oleic and stearic acid) from blanks and each other based on orthonasal as well as retronasal odor processing [12]–[15]. Taken together, these results indicate that humans are capable of detecting fatty acids by their odor. More important though, would be the ability to detect fat (and thus caloric) content in actual foods, as fat is an important determinant of palatability and reward [16]. Fat within food is mostly present in the form of triglycerides, and nearly always presented in combination with other odorous components that might influence or mask the olfactory perception of fat. Based on the data above, we hypothesize that, analogous to non-human animals, humans can detect fat content of food by our sense of smell alone.

In three behavioral experiments, we aimed to determine whether humans can detect fat based on the sense of smell alone, using a more natural setting, namely milk samples containing varying ecologically relevant percentages of fat. In Experiment 1, we determined whether humans are able to discriminate between fat content in a natural product based on their sense of smell alone in a North American sample (USA). In Experiment 2, we replicated the experiment in a different population (the Netherlands) where the average daily consumption per capita of milk as a beverage exceeds the US daily consumption by 31% [17]. Recent studies have suggested that sensitivity for fat taste is associated with BMI and habitual fat intake [6], [18], possibly due to exposure effects. Therefore, in Experiment 3, we determined whether BMI and habitual fat intake, this time assessed on the individual level, modulated individual’s ability to detect the odor of fat.

## Materials and Methods

### Ethics Statement

All participants provided written informed consent prior to participation and all aspects of the study were approved by the Institutional Review Board at the University of Pennsylvania.

### Experiment 1

**Participants.** A total of 30 participants (mean age 27.3±4.2 years; 14 men; mean BMI 23.1±3.1 kg/m^2^) recruited from the greater Philadelphia area (PA, USA) participated in the study. All participants were healthy, non-smoking, normosmic (as determined by the 16-item odor identification part of the Sniffin’ Sticks [19]), not pregnant or lactating, nor being lactose intolerance or having milk-related allergies. Subjects were asked not to eat or drink anything other than water one hour prior to testing, nor wear any scented products on the day of testing.

**Stimuli.** Odor stimuli were created from milk powder (FrieslandCampina, the Netherlands) rather than fresh milk to limit the potential influence various farm practices might have on the different milk samples. The experiments were performed with three different milk stimuli with varying fat content. The skim (S) and fat (F) samples were prepared by mixing 1 gram of 1.25% or 28% milk powder (Friesland Campina), respectively, with 10 ml of water. The medium (M) sample was made from a mix of 50% S sample and 50% F sample. This yielded fat percentages of 0.125%, 1.46%, and 2.8% for the S, M, and F samples, respectively.

**Study procedure.** Before the odor discrimination test, participants rated the perceived intensity and pleasantness of each of the three milk samples (S, M, & F) on a 100 mm visual analog scale (VAS). The intensity scale ranged from ‘not perceivable’ on the left to ‘extremely intense on the right’, and the pleasantness scale ranged from ‘unpleasant’ to ‘pleasant’. One participant did not complete these ratings. Participants were subsequently blindfolded and presented with a three-alternative, forced-choice odor discrimination test paradigm without feedback, consisting of a total of 27 milk sample triplets with 9 repetitions of each test combination (SSF, SSM, and FMM) using an inter-trial interval of approximately 30s between each triplet. Each trial consisted of three bottles, of which two contained the same milk sample, and one a different milk sample. Participants had to smell the bottles and choose the odd one out. Presentation order of the odor triplets was randomized, but the same for all participants. All milk samples were presented in 100 ml amber glass bottles, containing a total of 10 ml of liquid each with no visual markers.

**Data analysis.** The number of correct trials was summed up and converted to a percentage correct score. These percentages were analyzed using a one-sample t-test against chance level (33.3%) for the separate triplet combinations, as well as a sum score of all trials. Separate repeated measures ANOVAs were used to analyze possible differences in intensity, pleasantness, and discrimination ability between the various combinations.

### Experiment 2

**Participants.** A total of 18 healthy participants (mean age 22.1±1.2 years; 6 men; mean BMI 22.7 ±3.1 kg/m2) from the Wageningen area (the Netherlands) participated in the study. The same inclusion criteria and instructions as provided in Experiment 1 were applied.

**Stimuli.** A gas chromatography mass spectrometry (GC-MS) analysis indicated that the skim and fat milk samples used in Experiment 1 had minute differences in vitamin content. Although there is no known olfactory detection mechanism for vitamins, new batches of skim and fat milk powder with certified identical contents (by means of GC-MS) were used in Experiment 2. Odor stimuli were created from this new batch of milk powder (FrieslandCampina, the Netherlands) using an identical method as used in Experiment 1, except for a slight difference in fat concentration (skim powder: 1.25%, fat powder 26% fat percentage, yielding liquid samples with fat percentages of 0.125%, 1.36%, and 2.6% for the S, M, and F samples, respectively).

**Study procedure.** Before the odor discrimination test, participants rated the perceived pleasantness of each of the three milk samples (S, M, and F) on a 100 mm VAS. Participants were then presented with a total of 18 milk sample triplets using three repetitions of each unique combination: SSM, MMS, MMF, FFM, FFS, and SSF; this adjustment of stimuli in comparison to Experiment 1 was performed to counteract a potential target uniqueness effect; in other words, to counteract the possibility that one of the three categories is easier to identify among the others when being a target based on unrelated aspects that makes it an ideal target within a discrimination task. All other aspects were identical to what is described for Experiment 1 above.

**Data analysis.** Corresponding triplets were added together (SSM+MMS, SSF+FFS, MMF+FFM), resulting in three odor combinations; SM, SF and MF. Data were then analyzed as described above for Experiment 1. Additionally, to compare possible differences in discrimination ability between the two populations (Experiment 1 and Experiment 2), independent t-tests were done, for the separate triplet combinations, as well as for a sum score of all trials (percentage correct).

### Experiment 3

**Participants.** A total of 60 participants from the greater Philadelphia area (PA, USA) participated in Experiment 3. In addition to the normal-weight subject pool, the recruitment ad specifically asked for overweight individuals to apply for the study, yielding 30 normal weight participants (BMI between 18.5–25.0 kg/m2, mean BMI 22.5±1.8 kg/m^2^; mean age 25.0±3.7 years; 15 men) and 30 overweight participants (BMI > 27 kg/m^2^, mean BMI 35.6±8.4 kg/m^2^; mean age 30.6±7.2 years; 12 men). One participant was an outlier in terms of excessive BMI (70.5 kg/m^2^); we will present the main results on discrimination performance with and without this individual (results remain similar), while excluding this participant from the (secondary) analyses on pleasantness and intensity ratings, and dairy consumption. The same inclusion criteria and instructions as provided in Experiment 1 and 2 were applied.

**Stimuli.** The odor stimuli and preparation were identical to what is described for Experiment 2 above.

**Study procedure.** A Detecto weight beam physician scale (Detecto, Webb City, MO, USA) was used to determine each participant’s height and weight to calculate BMI (kg/m^2^). Then, participants rated the perceived intensity and pleasantness of each of the three milk samples (S, M, and F) on a 100 mm VAS. The odor discrimination task for the three samples was identical as described above for Experiment 2. At the end, participants completed a questionnaire about their habitual dairy product consumption (see File S1).

**Data analysis.** To analyze the results of the dairy consumption questionnaire, a product nutrient database was created with the help of the USDA National Nutrient Database for Standard Reference [20]. With this database, questionnaire responses were converted into total amount of milk (full and reduced) consumed per day (in grams and kcal) and total amount of fat from dairy consumed per day (grams). Data was then analyzed as described above for Experiment 2. Additionally, independent-sample t-tests were performed to analyze differences between groups (normal-weight, overweight) for discrimination sum score. Repeated measures ANOVAs were used to analyze the effect of BMI and dairy consumption on discrimination ability.

## Results

### Experiment 1

**Figure 1:**
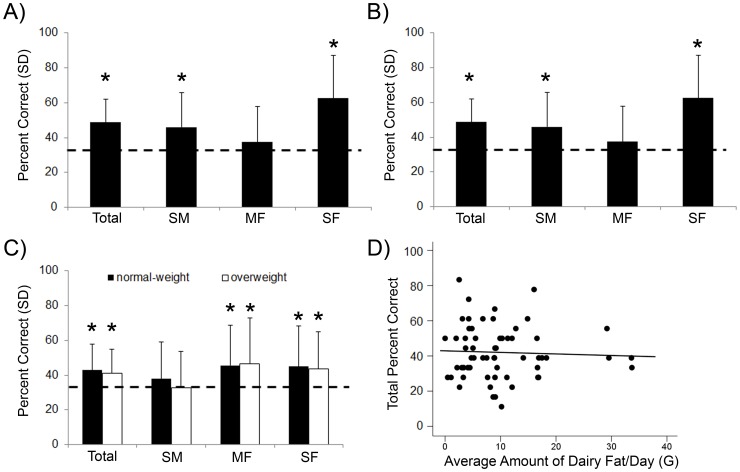
Mean percentage correct discrimination for each stimulus triplet. In all graphs: Error bars denote standard deviation, and stars above bar denotes results significantly different from expected chance performance (33.3%, p <.05). S  =  skim milk, M  =  medium milk, F  =  fat milk. See Methods section for further details regarding fat percentage. SM  =  discriminating between skim and medium milk; MF  =  discriminating between medium and fat milk; SF  =  discriminating between skim and fat milk. Dotted line in panel A, B, & C indicates expected chance performance (33.3%). **A**) Results of Experiment 1 in a North-American population. **B**) Results of Experiment 2 in a Dutch population. **C**) Results of Experiment 3 including normal-weight (black bars) and overweight individuals (white bars). **D**) Relationship between total discrimination performance and average daily dairy fat intake (in grams). Solid line in graph represents the regression line.

Overall, participants were able to discriminate between the different milk samples (mean total percentage correct  =  48.6, t(29) = 6.27, p <.001) significantly better than expected chance level (33.3%). Moreover, subjects were significantly able to discriminate the skim milk from medium milk (mean percentage correct  =  45.9, t(29)  = 3.47, p  = .002), as well as skim milk from fat milk (mean percentage correct  =  62.6, t(29)  = 6.50, p <.001). However, participants were not able to discriminate the medium milk from fat milk (mean percentage correct  =  37.4, t(29)  = 1.10, p  = .282), see Figure 1A.

Participants rated the intensity and pleasantness of the three milk samples significantly different. With increasing fat content, the sample was rated as more intense and less pleasant (intensity ratings: F[2,56]  = 35.36, p <.001: mean rating ± SD skim 16.7±1.4, medium 34.8±20.5, fat 47.0±24.3; pleasantness ratings: F[2,56]  = 3.90, p  = .026: mean rating ± SD skim 51.5±14.8, medium 47.3±18.8, fat 44.0±24.4).

### Experiment 2

As demonstrated above for Experiment 1, participants were overall able to discriminate between the different milk samples significantly better than chance (mean total percentage correct  =  53.1, t(17)  = 5.18, p <.001, chance level 33.3%). Moreover, participants were significantly able to discriminate the skim milk from medium milk (mean percentage correct  =  57.4, t(17)  = 3.31, p  = .004), as well as skim milk from fat milk (mean percentage correct  =  64.8, t(17)  = 5.87, p <.001). Similar to Experiment 1, participants were not able to discriminate the medium milk from fat milk (mean percentage correct  =  37.0, t(17)  = .73, p  = .477), see Figure 1B. Moreover, there were no statistical differences in percentage correct discrimination between Experiment 1 and 2, neither for the sum score of all trials, nor for the separate triplet combinations (sum score, t(46)  = 1.03, p  = .31; SM triplets, t(46)  = 1.57, p  = .12; MF triplets, t(46)  = .06, p  = .95; SF triplets, t(46)  = .31, p  = .76).

Contrary to the finding in Experiment 1, participants did not rate the pleasantness of the three milk samples significantly different (F[2,34]  = 1.58, p  = .221): mean rating ± SD skim 39.3±23.1, medium 39.8±20.7, fat 32.9±15.1.

### Experiment 3

In a direct replication of the findings in Experiment 1 and 2, participants were able to discriminate between the different milk samples significantly better than chance (mean total percentage correct  =  42.1, t(59)  = 4.49, p <.001; excluding BMI outlier, mean total percentage correct  =  41.9, t(58)  = 4.36, p <.001; chance level 33.3%). Moreover, participants were significantly able to discriminate the medium milk from fat milk (mean percentage correct  =  46.7, t(59)  = 4.40, p <.001; excluding BMI outlier, mean percentage correct  =  46.0, t(58)  = 4.21, p <.001), as well as skim milk from fat milk (mean percentage correct  =  43.9, t(59)  = 3.51, p  = .001; excluding BMI outlier, mean percentage correct  =  44.4, t(58)  = 3.65, p  = .001), but not the skim milk from medium milk (mean percentage correct  =  35.6, t(59)  = .82, p  = .417; excluding BMI outlier, mean percentage correct  =  35.3, t(58)  = .72, p  = .475).

There was no significant difference between normal-weight and overweight participants in their olfactory fat discrimination performance; neither on the combined score of all samples (mean total percentage correct for normal-weight 42.8, for overweight 41.3, t(58)  = .38, p  = .707; excluding BMI outlier, mean total percentage correct for overweight 41.0, t(57)  = .45, p  = .707), nor for the separate combinations (F[2,116]  = .992, p  = .677; excluding BMI outlier, F[2,114]  = .315, p  = .730; Figure 1C).

Participants rated the intensity and pleasantness of the three milk samples significantly different (intensity ratings: F[2,114]  = 4.45, p  = .014; pleasantness ratings: F[2,114]  = 6.73, p  = .002; see Table 1). Overweight participants rated the intensity, but not the pleasantness, of the milk samples significantly lower than normal-weight subjects (F[1,57]  = 4.80, p  = .033; F[1,57]  = .47, p  = .496, respectively; Table 1).

**Table 1:** Mean ± SD ratings for intensity and pleasantness for all three milk samples in Experiment 3, for all participants combined, and for normal-weight and overweight participants separately.

Ratings (100 mm VAS) ± SD		All	Normal-weight	Overweight
Intensity	Skim	28.0±20.3	31.4±21.5	24.4±18.6
	Medium	26.1±21.2	29.8±20.5	22.2±21.6
	Fat	33.7±18.6	39.8±18.2	27.3±17.1
Pleasantness	Skim	43.8±17.4	46.9±14.5	40.6±19.8
	Medium	44.1±15.5	45.7±14.5	42.4±16.6
	Fat	37.0±19.0	36.1±20.3	37.9±18.0

There was no significant correlation between either total amount of milk consumed per day (in grams and kcal) or amount of fat from dairy consumed per day (in grams, Figure 1D) with the ability to discriminate between the milk samples varying in fat percentage (r = –.121, p  = .401; r = –.100, p  = .453; r = –.018, p  = .895; respectively). Moreover, there was no significant difference in dairy consumption between normal-weight and overweight subjects (total amount of milk consumed per day in grams, t(57)  = .39, p  = .699; total amount of milk consumed per day in kcal, t(57)  = .09, p  = .933; amount of fat from dairy consumed per day in grams, t(57)  = .03, p  = .973).

## General Discussion

In the present study, we aimed to establish the ability of olfactory fat detection in humans using an ecologically relevant setting (milk samples containing varying realistic percentages of fat). In a series of three behavioral experiments, using study populations from different cultures, we demonstrated for the first time that humans can discriminate between varying concentrations of fat in a food product, using only their sense of smell. Moreover, this ability was not related to differences in BMI or dairy fat consumption.

Previous studies have demonstrated that humans can discriminate high concentrations of long-chain fatty acids in vapor phase both retronasally and orthonasally [12]–[15]. We here extend these findings by demonstrating that humans are able to discriminate between minor differences in fat content within natural food sources containing other volatiles that might mask the perceptual information that fat might emit. Importantly, whereas previous studies have used high concentrations of pure long-chained fatty acids (e.g. [12]), or multicomponent emulsions that likely activate the intranasal trigeminal nerve endings [7], we can here demonstrate that minute concentrations of fat within a natural food source can be detected by odors alone. Participants were able to discriminate between skim, medium, and fat milk samples, with an overall accuracy of 40–55% correct trials in three consecutive experiments, a value that is significantly above the expected chance level (33.3%). The consistency between studies clearly suggests that humans have a functional olfactory detection system that allows us to detect fat content within natural food sources. These data are in line with several animal studies that highlight the role of olfaction in (the formation of) preference for many high fat foods [8]–[11].

For all three experiments, our data demonstrate that participants are significantly able to discriminate between the different milk samples based on the overall scores. Specifically, in all studies, discrimination between skim and fat milk was significantly above chance level, which corresponds to the largest difference in the percentage of fat. However, in Experiment 1 and 2, participants were unable to discriminate medium milk from fat milk and in Experiment 3, they were unable to discriminate skim milk from medium milk. Although the absolute difference in fat percentage between skim-medium, and medium-fat was the same (each differing about 1.24%), it might be the relative difference in fat content that is relevant for humans to detect and hence be able to perceptually discriminate between. The difference in fat percentage from skim to medium is a 10-fold increase, whereas the fat percentage only doubles from medium to fat milk. However, another plausible explanation is that the just-noticeable-difference (JND) for these volatiles is in the 1.24% range, thus rendering discrimination between the medium fat concentration and the two endpoints inherently difficult to perform. Future studies should aim to assess discrimination performance between larger ranges of fat contents to establish the JND for fat odor discrimination.

Interestingly, there was no statistical difference in the ability to discriminate between varying fat percentages between different populations (US and the Netherlands) differing in their milk consumption, as well as no individual correlation with either BMI or dairy (fat) consumption. This is in contrast to previous studies by Stewart and colleagues who demonstrated that fat taste sensitivity is associated with both lower fat intake and BMI [6], [18]. Our results suggest, however, that olfactory-based fat discrimination is a stable trait that does not directly depend on previous exposure or learned associations. Note, however, that it is likely that all participants in all three studies had prior experience with milk. We can therefore not explicitly rule out the possibility that previous exposure does have a significant effect on fat odor detection.

In Experiment 1, the intensity and pleasantness of the three milk samples were perceived significantly different; the samples were rated as more intense and less pleasant with increasing fat content. This was not, however, the case for Experiment 2 and 3. The minute differences in vitamin content that existed between the fat and skim milk sample in Experiment 1 might be a factor here. However, even if the small differences in vitamin content mediated the difference in perceptual ratings in Experiment 1, we do not believe that those differences accounted for the main discrimination results; both Experiment 2 and 3 yielded nearly identical discrimination performance results using different milk samples. Our result that the milk samples could be discriminated from each other does indicate there are perceptual differences between them. We hypothesize that perhaps the perceptual differences are not directly attributable to their intensity or pleasantness ratings, but rather originate from an inability to categorize a separate perceptual quality such as ‘creaminess’ that we did not ask for in our experiments. Limitations in alternatives that are perceived as relevant can lead participants to ‘dump’ their response into the categories that are available for them. This phenomenon of so-called halo dumping is well established and might explain the conflicting outcomes between experiments in respect of the perceptual ratings [21].

Dietary fats, or triglycerides, are not known to be volatile. It is therefore unlikely that it is the fat per se in the milk that is the direct odor source which is detected and discriminated; hence it is as yet undetermined what chemical signal the participants are picking up with their nose in the milk sample. However, triglycerides can act as reservoirs of volatile flavor, that is, they can act as carrier of other volatile components that humans can detect in a fat-dependent manner. Alternatively, they are known to interact with other ingredients in the milk, thereby altering the olfactory percept [22], [23]. On the other hand, as mentioned previously, Halpern and colleagues have demonstrated that humans can detect isolated vapor-phase fatty acids [12]–[15], and possibly, it is these fatty acids in the milk samples that mediate our demonstrated effect. Lastly, it might have been oxidation products from the fat that participants were detecting, which would vary tightly along with the fat concentration in the milk samples. Further studies should focus on determining the components/volatiles in milk responsible for this effect by means of gas chromatography mass spectrometry (GC-MS) to analyze chemical differences between the headspace of the samples, as well as conjointly and targeted sniff port analyses to identify specific chemical components mediating the demonstrated perceptual differences.

In conclusion, these data demonstrate that humans are able to discriminate between varying grades of fat, even when embedded within a milk odor. Interestingly, and in contrast to fat taste, this ability is not related to either BMI or dairy fat consumption, suggesting this is not a learned ability or dependent on nutritional traits. The demonstration that humans have a functional olfactory system specific for detecting levels of fat content warrant further explorations into this mechanism given its potential to aid in a general reduction of our fat intake.

## Supporting Information

File S1 **Questionnaire used to assess dairy consumption.** Supplementary information is available at PLOS ONE’s website.
(DOCX)
