# Supplementary material for: Detecting Fat Content of Food from a Distance: Olfactory-Based Fat Discrimination in Humans
Source: PLoS One. 2014 Jan 22;9(1):e85977. doi: 10.1371/journal.pone.0085977 (PMC3899094; doi:10.1371/journal.pone.0085977)
Supplement: File S1 — Questionnaire used to assess dairy consumption. Supplementary information is available at PLOS ONE’s website. (DOCX) [file pone.0085977.s001.docx]

**Supplemental Information**

**Questionnaire dairy consumption**

### Instructions

This questionnaire is about dairy products and will be used to make an estimation of the dairy products you consumed the last month. The following remarks are important and should be considered during the answering of the questionnaire.

- Answer each question as best you can. Estimate if you are not sure. A guess is better than leaving a blank.
- The questions cover the last month, this means the last 4 weeks. This includes both weekdays and weekends
- The products you drank or ate at birthdays, weddings, receptions, etc. should be taken into account as well.
- If you had a completely different diet than normal for example due to illness or holiday for the last month, consider the month before the last month to fill in the questions.
- If you don’t use a product at all, fill in ‘Not this month’. Always give an answer

1a. How often did you drink **milk as a beverage** (NOT in coffee, NOT in cereal)? *(Please do not include chocolate milk, hot chocolate and flavored milk or* yogurt*)*

- Not this month **(go to question 2)**
- 1 day per month or less
- 2-3 days per month
- 1 day per week
- 2-3 days per week
- 4-5 days per week
- 6-7 days per week

1b. Each day you drank **milk as a beverage**, how much did you usually drink?

- Less than 1 cup (8 ounces)
- 1-1.5 cups (8 to 12 ounces)
- More than 1.5 cups (12 ounces)

1c. How often was the milk **reduced-fat or fat-free**?

- Almost never or never
- About ¼ of the time
- About ½ of the time
- About ¾ of the time
- Almost always or always

2a. How often did you drink **chocolate milk as a beverage (including hot chocolate)**?

- Not this month **(go to question 3)**
- 1 day per month or less
- 2-3 days per month
- 1 day per week
- 2-3 days per week
- 4-5 days per week
- 6-7 days per week

2b. Each day you drank **chocolate milk as a beverage**, how much did you usually drink?

- Less than 1 cup (8 ounces)
- 1-1.5 cups (8 to 12 ounces)
- More than 1.5 cups (12 ounces)

2c. How often was the chocolate milk **reduced-fat or fat-free**?

- Almost never or never
- About ¼ of the time
- About ½ of the time
- About ¾ of the time
- Almost always or always

3a. How often did you drink **flavored milks as a beverage**?

- Not this month **(go to question 4)**
- 1 day per month or less
- 2-3 days per month
- 1 day per week
- 2-3 days per week
- 4-5 days per week
- 6-7 days per week

3b. Each day you drank **flavored milks**, how much did you usually drink?

- Less than 1 cup (8 ounces)
- 1-1.5 cups (8 to 12 ounces)
- More than 1.5 cups (12 ounces)

3c. How often was the flavored milk **reduced-fat or fat-free**?

- Almost never or never
- About ¼ of the time
- About ½ of the time
- About ¾ of the time
- Almost always or always

4a. How often did you drink **yogurt as a beverage?**

- Not this month **(go to question 5)**
- 1 day per month or less
- 2-3 days per month
- 1 day per week
- 2-3 days per week
- 4-5 days per week
- 6-7 days per week

4b. Each of the days you drank **drink yogurt**, how much did you usually drink?

- Less than 1 cup (8 ounces)
- 1-1.5 cups (8 to 12 ounces)
- More than 1.5 cups (12 ounces)

4c. How often was the yoghurt **reduced-fat or fat-free**?

- Almost never or never
- About ¼ of the time
- About ½ of the time
- About ¾ of the time
- Almost always or always

5a. Do you consume **milk with cereals**?

- Not this month **(go to question 6)**
- 1 day per month or less
- 2-3 days per month
- 1 day per week
- 2-3 days per week
- 4-5 days per week

5b. Each time **milk was added to your cold cereal,** how much was usually added?

- Less than 1 cup (8 ounces)
- 1-1.5 cups (8 to 12 ounces)
- More than 1.5 cups (12 ounces)

5c. How often was the milk **reduced-fat or fat-free**?

- Almost never or never
- About ¼ of the time
- About ½ of the time
- About ¾ of the time
- Almost always or always

6a. How often did you eat **yogurt**?

- Not this month **(go to question 7)**
- 1 day per month or less
- 2-3 days per month
- 1 day per week
- 2-3 days per week
- 4-5 days per week
- 6-7 days per week

6b. Each time you ate **yogurt**, how much did you usually eat?

- Less than ½ cup or less than 1 container
- 0 ½ to 1 cup or 1 container
- 0 More than 1 cup or more than 1 container

6c. How often was the yogurt you ate **reduced-fat or fat-free**?

- Almost never or never
- About ¼ of the time
- About ½ of the time
- About ¾ of the time
- Almost always or always

7a. How often did you eat **cottage cheese**?

- Not this month **(go to question 8)**
- 1 day per month or less
- 2-3 days per month
- 1 day per week
- 2-3 days per week
- 4-5 days per week
- 6-7 days per week

7b. Each time you ate **cottage cheese**, how much did you usually eat?

- Less than 1 cup (8 ounces)
- 1-1.5 cups (8 to 12 ounces)
- More than 1.5 cups (12 ounces)

7c. How often was the cottage cheese you ate **reduced-fat or fat-free**?

- - Almost never or never
  - About ¼ of the time
  - About ½ of the time
  - About ¾ of the time
  - Almost always or always

8a. How often did you eat **pudding or custard**?

- Not this month **(go to question 9)**
- 1 day per month or less
- 2-3 days per month
- 1 day per week
- 2-3 days per week
- 4-5 days per week
- 6-7 days per week

8b. Each time you ate **pudding or custard,** how much did you usually eat?

- Less than 1 cup (8 ounces)
- 1-1.5 cups (8 to 12 ounces)
- More than 1.5 cups (12 ounces)

8c. How often was the pudding or custard you ate **reduced-fat or fat-free**?

- - Almost never or never
  - About ¼ of the time
  - About ½ of the time
  - About ¾ of the time
  - Almost always or always

9a. How often did you eat **sour cream**?

- Not this month **(go to question 10)**
- 1 day per month or less
- 2-3 days per month
- 1 day per week
- 2-3 days per week
- 4-5 days per week
- 6-7 days per week

9b. Each time you ate **sour cream,** how much did you usually eat?

- Less than 1 tablespoon
- 1 to 3 tablespoon
- More than 3 tablespoons

9c. How often was the sour-cream you ate **reduced-fat or fat-free**?

- - Almost never or never
  - About ¼ of the time
  - About ½ of the time
  - About ¾ of the time
  - Almost always or always

10a. How often did you eat **cheese** (including low-fat; including on cheeseburgers or in sandwiches or subs)?

- Not this month **(go to question 11)**
- 1 day per month or less
- 2-3 days per month
- 1 day per week
- 2-3 days per week
- 4-5 days per week
- 6-7 days per week

10b. Each time you ate **cheese**, how much did you usually eat?

- Less than ½ ounce or less than 1 slice
- ½ to 1½ ounces or 1 slice
- More than 1½ ounces or more than 1 slice

10c. How often was the cheese you ate **reduced-fat or fat-free**?

- - Almost never or never
  - About ¼ of the time
  - About ½ of the time
  - About ¾ of the time
  - Almost always or always

11a. How often did you eat **cream cheese?**

- Not this month **(go to question 12)**
- 1 day per month or less
- 2-3 days per month
- 1 day per week
- 2-3 days per week
- 4-5 days per week
- 6-7 days per week

11b. Each time you ate **cream cheese**, how much did you usually eat?

- Less than 1 tablespoon
- 1 to 3 tablespoon
- More than 3 tablespoons

11c. How often was the cream cheese you ate **reduced-fat or fat-free**?

- - Almost never or never
  - About ¼ of the time
  - About ½ of the time
  - About ¾ of the time
  - Almost always or always

12a. How often did you eat **whipped cream?**

- Not this month **(go to question 13)**
- 1 day per month or less
- 2-3 days per month
- 1 day per week
- 2-3 days per week
- 4-5 days per week
- 6-7 days per week

12b. Each time you ate **whipped cream**, how much did you usually eat?

- Less than 1 tablespoon
- 1 to 3 tablespoon
- More than 3 tablespoons

12c. How often was the whipped cream you ate **reduced-fat or fat-free**?

- - Almost never or never
  - About ¼ of the time
  - About ½ of the time
  - About ¾ of the time
  - Almost always or always

13a. How often do you drink **coffee or tea with milk**?

- Not this month **(last question, thank you for completing this questionnaire)**
- 1 day per month or less
- 2-3 days per month
- 1 day per week
- 2-3 days per week
- 4-5 days per week

13b. Each time **milk was added to your coffee or tea**, how much was usually added?

- Less than 1 tablespoon
- 1 to 3 tablespoon
- More than 3 tablespoons

13 c. How often was the milk **reduced-fat or fat-free**?

- Almost never or never
- About ¼ of the time
- About ½ of the time
- About ¾ of the time
- Almost always or always

**This is the last question, thank you very much for completing this questionnaire**

*Based on the Diet History Questionnaire, Version 2.0. National Institutes of Health, Applied Research Program, National Cancer Institute,2010. & the Dutch Dairy Food Frequency Questionnaire, Wageningen University, Department Human Nutrition, 2005*
